# Children With Tetralogy of Fallot in an Urban Centre in Africa

**DOI:** 10.15171/jcvtr.2015.36

**Published:** 2015-12-01

**Authors:** Barakat Adeola Animasahun, Akpoembele Deborah Madise-Wobo, Samuel I Omokhodion, Olisamedua Fidelis Njokanma

**Affiliations:** ^1^ Department of Paediatrics and Child Health, Lagos State University College of Medicine, Ikeja, Lagos, Nigeria; ^2^ Department of Paediatrics, Lagos State University Teaching Hospital, Ikeja, Lagos, Nigeria; ^3^ Department of Paediatrics and Child Health, University College Hospital, badan, Oyo State, Nigeria

**Keywords:** Tetralogy, Fallot, Profile, Nigeria, Africa

## Abstract

***Introduction:*** There is a dearth of literature on tetralogy of fallot (TOF) in children in Sub-Saharan Africa. This study up aims to describe the prevalence, clinical profile and associated cardiac anomaly of children diagnosed with TOF documented over an eight year period in a tertiary hospital in South Western Nigeria.

***Methods:*** A prospective review of all consecutive cases of TOF diagnosed with echocardiography at the Lagos State University Teaching Hospital (LASUTH) between January 2007 and December 2014. Data were analyzed using SPSS version 20. Tables and charts were used to depict those variables. Descriptive statistic are presented as percentages or means and standard deviation. Means of normally distributed variables were compared using the student *t* test and proportions using chi-square test. Skewed distribution were analyzed using appropriate non-parametric tests. Level of significance set at *P* < 0.05.

***Result:*** The prevalence of TOF among children presenting at LASUTH at the study period was 4.9 per 10 000 while its prevalence among those with congenital heart disease was 16.9%. There was a male predominance and most children presented within 1-5 years of age. Chromosomal abnormalities such as Down syndrome, Turners syndrome and CATCH 22 syndrome were documented in some subjects. Some of the subjects had atypical presentation.

***Conclusion:*** TOF is as common in Nigeria as other parts of the world, there is a need to established cardiac centers to salvage these children. Collaboration from developed countries will be helpful in this resource limited region.

## Introduction

Tetralogy of fallot (TOF) is the most common cyanotic heart lesion beyond the neonatal period^1^ and it accounts for a third of all congenital heart disease in patients less than 15 years of age.^2^ It occurs in 10% of all congenital heart disease.^3^ The prevalence of TOF is approximately 3.9 per 10 000 live births in the United States^4^ and 10%-26.2% of all congenital heart diseases in Nigeria.^4-11^

Diagnosis of TOF is confirmed with echocardiography.^3^ Definitive diagnostic features and other associated cardiac abnormalities can be identified. In advanced countries, diagnosis can be made in as early as 12 weeks of gestation with fetal echocardiopgraphy.^12,13^

Management is both medical and surgical and depends on the degree and type of right ventricular outflow tract obstruction and the centre’s protocol.^3^ There is a dearth of literature on TOF in children in Sub-Saharan Africa. This study aim to describe the prevalence, clinical profile and associated cardiac anomaly of children diagnosed with TOF documented over an eight year period in a tertiary hospital in South Western Nigeria.

## Materials and Methods

Prospective and cross-sectional, involving all cases of TOF diagnosed with echocardiography at the Lagos State University Teaching Hospital,( LASUTH) between January 2007 and December 2014. The center is a tertiary hospital in South Western Nigeria. The Hospital receives referral from the South Western region and is the largest center that admits pediatric cases.

A pediatric cardiologists reviewed all the subjects referred for evaluation. A GE Vivid Q echocardiography machine with reference number 14502 WP SN 2084 with appropriate sized probes was used throughout the study period and the pediatric cardiologists performed the echocardiography on all the study subjects. Definitive diagnosis of TOF was made with echocardiography.^3^

The data were analyzed using SPSS version 20. The prevalence of TOF was calculated from all children who presented in the hospital as in-patient and out-patient during the study period. The prevalence of TOF was also calculated amongst those with congenital heart lesions and those with cyanotic congenital heart disease. Tables and charts were used to depict those variables. Descriptive statistic are presented as percentages or means and standard deviation. Means of normally distributed variables were compared using the student *t*-test and proportions using chi-square test. Skewed distribution were analyzed using appropriate non-parametric tests. Level of significance set at *P *< 0.05

## Results

### Prevalence of Tetralogy of Fallot

A total of 155 patients with echo diagnosis of TOF were documented between January 2007 and December 2014 with a total of 315 150 patients seen as out-patient and in-patient at the study centre during the study period hence the prevalence of TOF amongst the children who were seen in the hospital during the study period was 4.9 per 10 000. A total of 983 had congenital heart disease while 311 of the 983 patient with congenital heart disease had cyanotic congenital heart disease therefore, TOF accounted for 15.8% and 49.8% of congenital heart disease and cyanotic congenital heart disease respectively. Table 1 depicts the prevalence of TOF in the study subjects. Table 2 illustrates the prevalence of TOF in other countries.

**Table 1:** Yearly Prevalence of TOF at the Study Center

**Year**	**CHD (n)**	**CCHD (n)**	**TOF (n)**	**% of TOF in CHD**	**% of TOF in CCHD**
2007	87	22	13	14.9	59.1
2008	119	29	10	8.4	34.5
2009	90	23	18	20	86.9
2010	103	36	12	11.6	33.3
2011	153	48	27	17.6	56.3
2012	143	47	26	18.2	55.3
2013	180	65	27	33.7	41.5
2014	108	41	12	11.1	29.3
Total	983	311	155	15.8	49.8

Abbreviations‏: CHD, congenital heart disease; CCHD, cyanotic congenital heart disease; TOF; tetralogy of Fallot.

**Table 2:** Country Prevalence of TOF in CHD

**City/Country**	**Author**	**Year** ^a^	**Percent** ^b^
Malawi	Kennedy and Miller^14^	2013	17.9
Zimbabwe	Bannerman et al^15^	1998	19.6
Iran	Rahim et al^16^	2008	16.9
Iraq	Khadim and Issa^17^	2009	12.6
India	Kapoor and Gupta^18^	2008	4.6
Sudan	El Hag^21^	1994	4.2
Saudi Arabia	Alabdulgader^22^	2006	3.5
Qatar	Robida et al^23^	1997	5.1
Denmark	Laursen^24^	1980	5.8
Oman	Subramanyan et al^25^	1998	9.6
Taiwan	Shann^26^	1969	18.1
Canada	Rose et al^27^	1964	8.1

Abbreviations‏: CHD, congenital heart disease; TOF; tetralogy of Fallot. ^a^Year of publication‏. ^b^Percentage of TOF in all CHD‏.

### Clinical Presentation

The male to female ratio was 1.7:1. The mean age of the children was 51.6 ± 52.5 (months). Table 3 describes the age groups of the patients. The most common indication for cardiac evaluation in the study subjects was cyanosis with a suspicion of a cyanotic congenital heart disease. 110 (71%) children were clinically cyanosed on presentation. Record of oxygen saturation was documented with pulse oximeter in 89 subjects. 69 of the 89 (77.5%) of the pulse oximeter records had evidence of cyanosis and 63/69 (91%) of the pulse oximeter recordings of cyanosis were truly cyanosed.

**Table 3:** Age Groups of the Subjectsa

**Age Groups**	**Male**	**Female**	**Total**	**Percent**
≤ 6 months^b^	12	8	20	13.8
> 6 months -12 months	11	9	20	13.8
>12 month-5 years	36	25	61	42.1
> 5-10 years	13	1	14	9.6
Above 10 years	18	12	30	20.7
Total	90	55	145	100

^a^ The age of four males and five females were missing. The sex and age of one subject was not documented. ^b^ *P* value = 0.17, x^2^= 6.4.

### Association Syndromes

**Figure 1:**
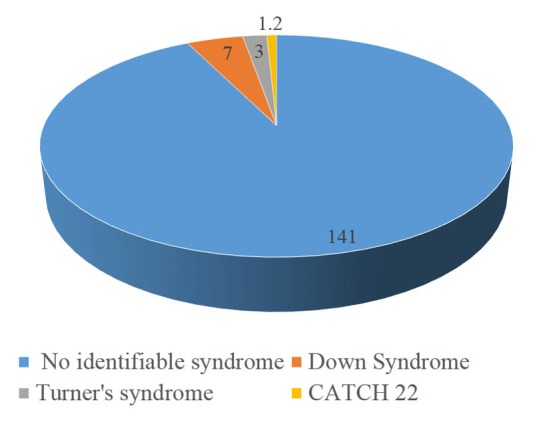
Associated Chromosomal Anomalies/Syndromes.

However, Down syndrome was documented on 7 subjects, 3 had Turners syndrome and one had CATCH 22. This is shown in Figure 1.

## Discussion

The prevalence of TOF in children in the present study is 4.9 per 10 000 children. This prevalence rate is a hospital based result and thus may not represent the prevalence in the general population. Prevalence rate of TOF have been documented in relation to live birth. A prevalence of 3-6 infants per 10 000 live birth have been reported by Bhmji.^2^ Population prevalence rate is not known in Sub-Saharan Africa.

The general prevalence of TOF amongst all congenital heart disease is 10%.^2,3^ Different researchers have reported varying rates depending on the region of study. In the present study, TOF was recorded in 15.8% of all congenital heart diseases. Previous studies in Nigeria have documented rates between 10% and 26.2% in all congenital heart diseases.^4-11^ Prevalence rate of TOF in all congenital heart diseases in other countries within Africa is between 10 and 19.6%.^14-16^ Similarly the authors in Africa had different methods, study subjects, and duration. The prevalence rate in other centers in Iran^17^ and Iraq^18^ is 16.9% and 12.6%, respectively which is similar with reports in Africa. In India, different authors have recorded rates between 4.6% and 5.5%.^19,26^

There was a male predominance of TOF in the present study with a male to female ratio of 1.7:1. This finding is similar with other earlier reports.^18,19,26^ TOF being a cyanotic congenital heart disease, 71% of our subjects presented with cyanosis. Pulse-oximeter is a vital tool in evaluation of patients with congenital heart disease, we were able to document cyanosis using pulse oximeter in up to 91% of the subjects who were cyanosed. Few patients, 5 (3.2%) presented with atypical presentation like gangrene of the right hand and forearm, stroke, cerebral abscess and congestive cardiac failure. Onset of clinical manifestation in patients with TOF depends on the severity of right ventricular outflow tract obstruction.^3^ Patients may present in neonatal period when there is severe obstruction to right ventricular outflow. The youngest patient was a 2 days old female who was part of a conjoint twin with TOF and pulmonary atresia. Two-thirds of the patients were under 5 years at diagnosis and 60.4% of those were between 1 and 5 years. One-third of the subjects were more than 5 years at presentation. The mean age of the subjects at presentation was 51.6 ± 52.5 in months (4.3 ± 4.4 in years). This mean age is almost similar to that reported by Kennedy and Miller^14^ in Malawi. Other authors have presented earlier age at diagnosis of TOF.^26^ However diagnosis of TOF and other congenital heart diseases are usually made between 1-5 years of age, especially in resource poor countries.^13,18,27^ Possible reasons for the trend of late presentation and diagnosis includes difficulty in assessing specialized care, poverty and poor health seeking behaviour.^12,13,17^

Chromosomal anomalies have been documented in up to 25% of patients with TOF, the commonest being trisomies and 22q11.2 micro deletion syndromes.^3-5^ We documented in this study 11 (7.1%) patients with TOF and chromosomal anomalies. There were 7 (4.5%) patient with Down syndrome, 3 (1.9%) of Turners syndrome and 1 (0.65%) case of CATCH 22 syndrome. Risk of recurrence rate for TOF is 3%,^3^ in this study we had two cases of TOF from the same family, a younger and other sibling.

Patients with TOF may have other associated cardiac defects. In this study, we documented the other associated cardiac lesions in our subjects with TOF. The most common was pulmonary atresia followed by atrial septal defect (ASD). All the patients with ASD had secundum ASD.

## Conclusion

We report a prevalence of 4.9 per 10 000 children in this hospital base study. The prevalence of TOF in congenital heart disease is 15.8% and this was similar to other studies within Nigeria and the sub-region. There was a male predominance and most children presented within 1-5 years of age. Chromosomal abnormalities such as Down syndrome, Turners syndrome and CATCH 22 syndrome were documented in some subjects. Some of the subjects had atypical presentation. TOF is as common in Nigeria as other parts of the world, there is a need to established cardiac centers to salvage these children. Collaboration from developed countries will be helpful in this resource limited region.

## Acknowledgements

We gratefully acknowledge the children who participated in this study and their parents.

## Ethical Issues

Ethical clearance was obtained from the ethical committee of the hospital.

## Competing Interests

None to be declared.
